# Endangered edible orchids and vulnerable gatherers in the context of HIV/AIDS in the Southern Highlands of Tanzania

**DOI:** 10.1186/1746-4269-5-41

**Published:** 2009-12-18

**Authors:** Joyce FX Challe, Lisa Leimar Price

**Affiliations:** 1 Department of Plant Sciences (CWE), Wageningen University, PO Box 430, 6700AK Wageningen, the Netherlands; 2 Department Social Sciences (SCH). Wageningen University, PO Box 8060, 6700 DA Wageningen, the Netherlands

## Abstract

**Background:**

Tanzania is a wild orchid biodiversity hotspot and has a high prevalence of HIV/AIDS. The wild orchids in the study are endemic and protected by the Convention on International Trade in Endangered Species. Every year, however, between 2.2 and 4.1 million orchid plants consumed in Zambia are estimated as originating from Tanzania. This research examines the differences between HIV/AIDS wild edible orchid gatherers and non-HIV/AIDS gatherers with regards to the frequency of gathering, salience in naming the various orchids, gathering knowledge acquisition and perceptions regarding the current state of abundance of the edible species.

**Methods:**

Data was collected through interviews with 224 individuals in the Makete District of Tanzania close to the boarder of Zambia. Free-listings were conducted and Sutrup's Cultural Significance Index (CSI) constructed. The independent t-test was used to compare the differences in gathering frequencies between affected and non-affected gatherers. A multiple comparison of the 4 subgroups (affected adults and children, and non-affected adults and children) in gathering frequencies was done with a one way ANOVA test and its post hoc test. To examine the difference between affected and non-affected gatherers difference in source of gathering knowledge, a chi square test was run.

**Results:**

Forty two vernacular names of gathered orchid species were mentioned corresponding to 7 botanical species belongs to genera *Disa, Satyrium, Habenaria, Eulophia and Roeperocharis*. Ninety-seven percent of HIV/AIDS affected households state that orchid gathering is their primary economic activity compared to non-HIV/AIDS affected households at 9.7 percent. The HIV/AIDS affected gathered significantly more often than the non-affected. AIDS orphans, however, gathered most frequently. Gatherers perceive a decreasing trend of abundance of 6 of the 7 species. Gathering activities were mainly performed in age based peer groups. The results revealed a significant difference between affected and non-affected individuals in terms of their source of gathering knowledge.

**Conclusions:**

HIV/AIDS is related to increased reliance on the natural environment. This appears even more so for the most vulnerable, the AIDS orphaned children followed by HIV/AIDS widows.

## Background

One impact of HIV/AIDS has been the creation of a large number of orphaned children. It is estimated that 12 million children in African countries are currently HIV/AIDS orphans and that the number of AIDS orphaned children under the age of 18 will increase to more than 14 million by 2015 [1,2]. This paper reports on a study which examines the collection of wild edible orchids by HIV/AIDS status in the southern highlands of Tanzania. Three rural villages among three tribes (Kinga, Bena and Wanji) were used as samples for the research. The research area has a high prevalence of HIV/AIDS and a large number of AIDS orphans [3].

While gathering wild plants for both consumption and market sale is quite common among farming communities there is evidence that households affected by HIV/AIDS may utilise wild food plants as one of their survival strategies [4-7]. Such increased reliance upon the bounty of nature brings intensified interaction with the ecosystem surrounding the affected community.

**Figure 1:**
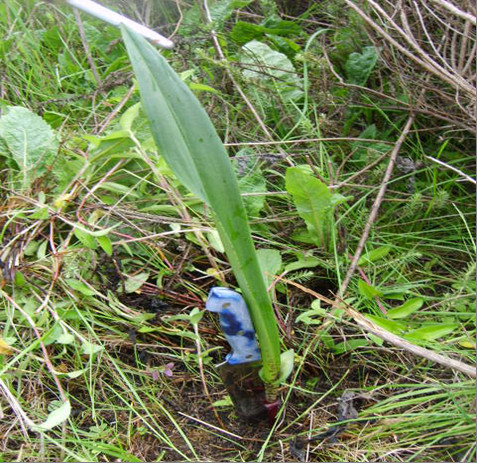
***Satyrium atherstonei *Rchb.f, edible orchid in the wild leaning on a knife**.

**Figure 2:**
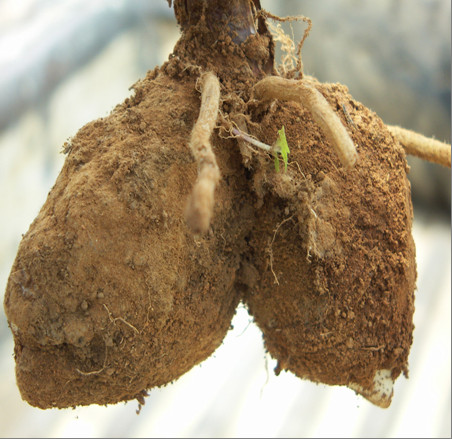
**Edible orchid tuber**. Non-consumable old depleted tuber depicted on left with newly formed consumable tuber on the right.

**Figure 3:**
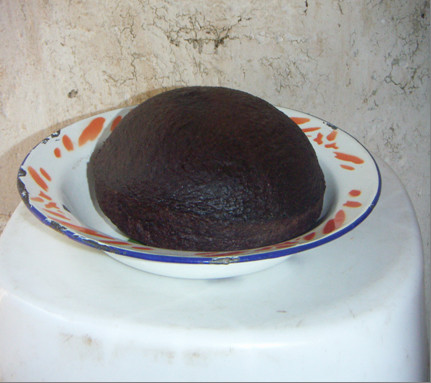
***Chikanda*, a meatless sausage produced from edible orchids**.

In the context of this study focusing on wild orchid gathering (Figure 1 and Figure 2), however, the necessities of HIV/AIDS are not all that need consideration. Significantly, there is a pre-existing large market for edible orchid tubers. These two aspects combined bring incredible pressure to bear on edible orchids. Edible orchid tubers in the research area have traditionally been consumed as a midday snack food. This traditional pattern of use changed with the introduction of an international market for wild orchid tubers in southern African countries, with countries such as Zambia creating increased demand for Tanzanian orchid tubers. In Zambia, the tubers from the orchid genera *Disa, Habenaria *and *Satyrium *are the main ingredients of "*chikanda*" (Figure 3), a popular meatless sausage [8-10]. As edible species went into severe decline in Zambia, demands started being met from Tanzania. Between 2.2 and 4.1 million orchid plants consumed annually in Zambia are estimated as originating from Tanzania [11]. One orchid plant produces one edible tuber (roughly the size of one small Irish potato) and the entire plant is removed in the harvesting process. Ultimately, gathering pressure is considered to be the cause of decrease in the diversity of wild orchid species [12] as noted in other wild edible plants [13]. The wild orchid plants in the study are endemic and protected by the Convention on International Trade in Endangered Species of Wild Fauna and Flora [14] and are reported to be under threat of extinction due to unsustainable harvesting [11,12]. Such an increase in reliance on natural resources is likely to reduce the survival of the exploited species.

Understanding issues of over-exploitation as well as the sustainable gathering of non-timber forest products (NTFPs), including wild gathered orchids, requires not only that the interface of the ecological, biological and economic dimensions need to be taken into consideration, but that the socio-cultural dimension be taken into consideration as well [15]. Environmental knowledge, particularly surrounding food resources, is culturally important.

It is now well known that the illegal trade in wild edible orchids contributes to their further endangerment yet little is understood about the gatherers themselves. Despite several scholars noting the devastating impact that HIV/AIDS has on most households and wild edible plants, no quantitative or in-depth studies have been conducted to examine differences between HIV/AIDS affected and non-HIV/AIDS affected households in terms of wild edible orchid gathering. Therefore, the aim of this study is to investigate how HIV/AIDS affected gatherers differ from non-HIV/AIDS affected gatherers. The differences investigated in this paper include gathering frequency, salience in naming gathered wild orchids, gathering knowledge acquisition and perceptions of changes in abundance. Several questions are answered in this paper: Do HIV/AIDS affected wild edible orchid gatherers gather more frequently than non-HIV/AIDS affected gatherers? Do HIV/AIDS affected orphans gather more frequently than HIV/AIDS affected adults? Is the source of wild orchid gathering knowledge different for the different groups in the study (HIV/AIDS affected adults, AIDS orphans, non-affected adults, and non-affected children)? What are the most salient edible orchid species and what are the gatherer's perceptions regarding edible wild orchid abundance?

## Methods

### Research Site and Sample

**Figure 4:**
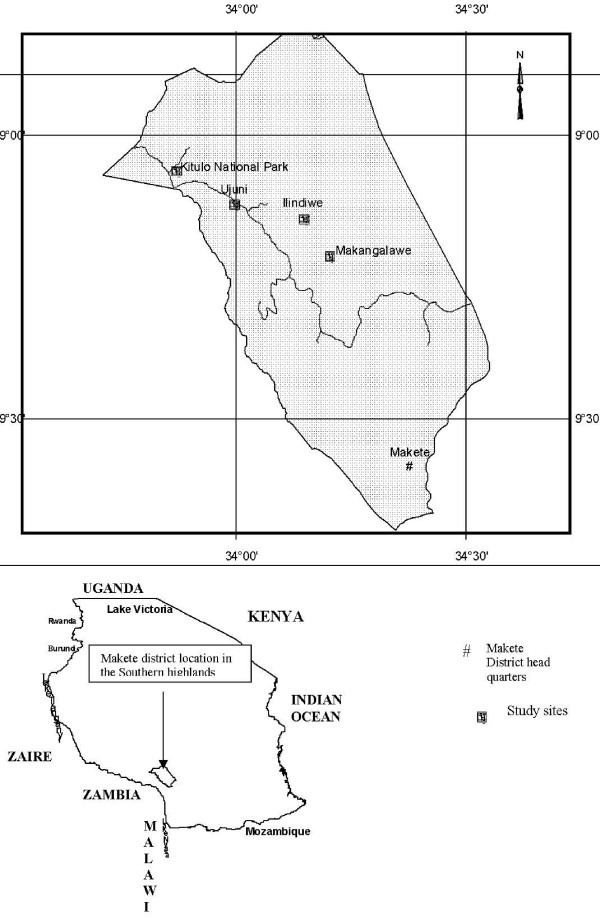
**Map of study area**.

Three villages were selected based on the communities having a prevalence of HIV/AIDS and practicing wild orchid gathering. The villages are located within Makete district in the southern highlands of Tanzania where wild orchids have a hospitable natural habitat. The Makete district, which is close to Zambia, is about 332 kilometres from the Iringa municipality, between 33°85' and 34°30' E and between 8°45 and 9°40' S (Figure 4). Makete district occupies an area of 5,800 square kilometres with a sparse population. In this district, agriculture is the mainstay of the economy with cash crops including pyrethrum and coffee, and food crops being maize, wheat, Irish potatoes, sweet potatoes, millet, sorghum, barley and various types of fruit. Livestock such as cattle, goats, sheep, chickens and pigs are also reared and some farmers are involved in tree planting for timber. Game reserves occupy 9,000 ha, natural forests 11,821 ha and plantation forests 7,313 ha. The area under cultivation is 24,459 ha, representing 20.3% of the total arable land [16].

**Figure 5:**
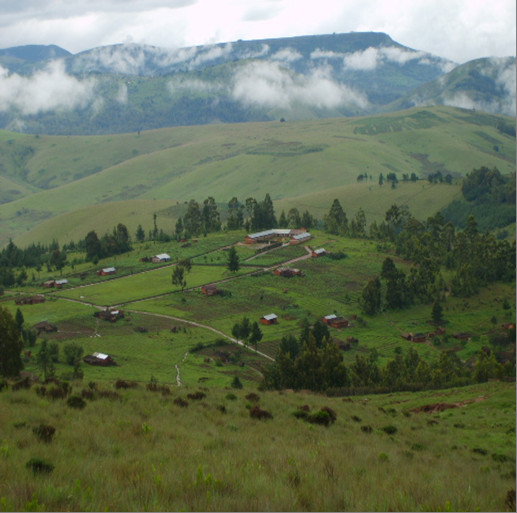
**Photo of the research area landscape**.

The land surface comprises undulating plains, mountains, grasslands and wetlands (Figure 5). Makete, as part of the vast montane grasslands, has diverse grassland species and perennial geophytes, including a significant number of terrestrial orchids [11]. Most wild gathered orchids grow primarily in areas without cultivation, usually on hillsides and in montane grasslands.

Informants for this study included two main categories, HIV/AIDS affected and non-affected. To obtain the sample population, a simple census interview questionnaire was given to 400 primary school children to fill in at home. The one page form included questions on head of household and other household members; presence or absence of orphans; awareness of orchid tuber gathering; involvement of household members in gathering orchid tubers; main gatherers in the household and presence of anyone in their household from outside the village staying with them for the purpose of gathering. From the children's returned forms, a list of households aware of and involved in orchid tuber gathering was obtained. One hundred and fifty two households were identified as engaging in orchid tuber gathering. There were 224 gatherers identified because some households had more then one gatherer. Of the gatherers sampled in this study, 111 live in HIV/AIDS affected households (including 80 orphaned children having at least one deceased parent) and 113 gatherers live in non-affected households (including 56 non-orphaned children).

### Data collection

Field observations conducted to determine the types of orchids present and species diversity that confirm the diversity of orchids in the study area has been reduced due to gathering pressure is reported elsewhere in detail [12].

Data used in this paper comes from a semi-structured in-depth questionnaire administered to 224 orchid tuber gatherers. The data selected for analysis includes the HIV-AIDS status, orphan status, wild orchids known, gathering patterns, perceived abundance of the species known to the informants, and source of orchid tuber gathering knowledge.

Embedded within the larger questionnaire was a free listing task where informants were asked to list the edible orchids they know. Free listing is argued to be among the most successful initial techniques for delimiting a domain of knowledge, especially when there is a need of uncovering the most salient items [17,18]. It is assumed that frequently mentioned items or species across informants and the mention of items at or close to the beginning of the lists implies an agreement on the relative cultural importance (salience) of the items. The free list data was analyzed using the two parameters of term frequency and mean position in the lists as developed by Sutrop [19] and successfully applied in the analysis of an aspect of HIV/AIDS orphan's ethnobiological knowledge in Benin [20]. Informants were asked about their perception regarding the current state of abundance relative to when they first started gathering. The estimation of abundance of species known to them was based on response options that included 1) decreasing abundance; 2) no change in abundance; and 3) increase in abundance. Gathering frequency data was given by self-report based on number of days per week spent gathering wild edible orchid tubers.

### Data analysis

Estimation of *the cognitive salience index (CSI) *was calculated using the formula S = F/(N mP)

F being the frequency of a term across the lists;

N being the total number of lists (subjects);

R_j _being the rank of a term in an individual list.

The mean position of a term (across the lists) is calculated as follows:

mP = (ΣR_j_)/F, hence, the cognitive salience index is rewritten as:

*CSI values range between 1 as most salient and 0 least salient*.

Student's t-test was used to compare gatherers from HIV/AIDS affected and non-affected households in orchid tuber gathering frequency per week, number of wild edible orchids known and number of gatherers per household. For multiple comparisons in gathering frequencies between gatherer categories (affected and non-affected adults and children), a one way ANOVA test (and its post hoc test) was run to test for statistical significance at the *p *< 0.05 level.

A chi square (*X*^2^), was employed to determine if there was a significant difference (*p *< 0.05) in the source of gathering knowledge between the two major categories of gatherers (HIV/AIDS affected and non-HIV/AIDS affected).

## Results

### Taxonomic diversity and vernacular names

Forty two vernacular names of gathered orchid species were mentioned by the three tribes in the study area. The Kinga tribe (cultural majority) [16] mentioned 39 vernacular names, followed by 14 names known by the Bena and 11 known by the Wanji tribe. The 42 ethno-species correspond to seven botanical species from one family, *Orchidaceae*. The seven botanical species belong to five genera: *Disa*, *Satyrium, Habenaria, Eulophia *and *Roeperocharis *(Table 1).

**Table 1:** Gathered edible orchid species, local names free listed and voucher specimen details

Wild orchid species	Local name	Voucher/Specimen (number) *
*Disa erubescens *Rendle	Makaha ga mlutu^ab^, Masekele^a^, Masekeni^a^,	1265
*Disa robusta *N.E. Br.	Liseku^ac^, Manene^a^, Masekeniyakizungu^a ^Mekundu^ac^, Vikubwa^a^, Vyekundu^a^,	1257
*Satyrium atherstonei *Rchb. f.	Ingingi^a ^Jike^a^Lidala^a^, Lisekejike^ab^, Lisekeni^c^, Lisekenilidala^ac^, Madala^a^, Masekenimadala^ab^, Numbunumbu^a^, Sidala^ab^, Visekenividala^ac^, Vijike^a^.	1327
*Habenaria xanthochlora *Schltr.	Mamkumungu^a^, Manseke^a^, Mansekemakubwa^a^, Mviringo^a^, Likose^abc^, Liseke^abc^.	1254
*Satyrium buchananii *Schtr.	Dochamua^ab^, Ligosi^ab^, Likosi^bc^, *Lisekedochamua*^ab^, Lisekedume^ab ^Lisekekiume^a^, Magosi^abc^, Masekenidume^a^, Masekeni magosi^ab^, Sisekeni sigosi^c^, Titisigosi^a^, Visekenivikhosi^abc ^Visekenivigosi^ac^.	1330
*Eulophia schweinfurthii *Kraenzl	Lisesa^a^	1323
*Roeperocharis wentzeliana *Kraenzl	Masekele^a^	

**Tribe (s)**: ^a ^Kinga, ^b ^Bena, ^c ^Wanji

Voucher specimen is on repository at the National Genetic Resource Herbarium, Arusha, Tanzania

### Wild orchid gatherers

**Figure 6:**
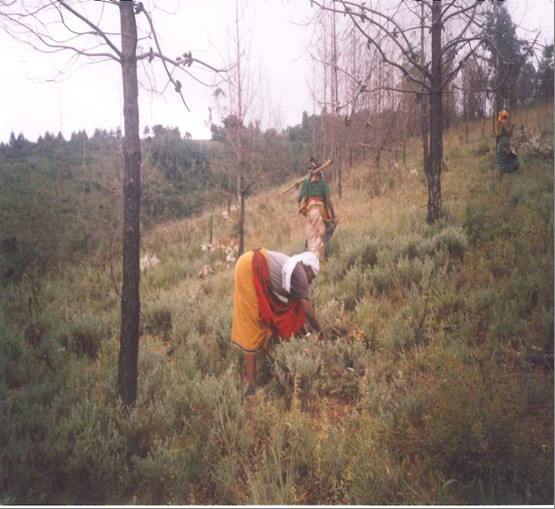
**Women gathering wild edible orchid tubers**.

**Figure 7:**
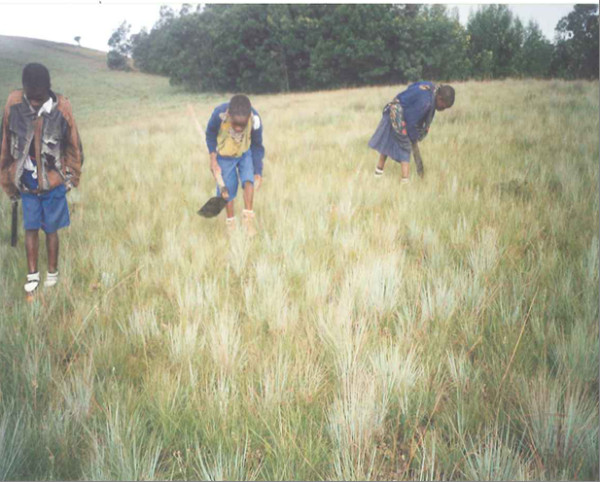
**AIDS orphans gathering wild edible orchid tubers**.

**Figure 8:**
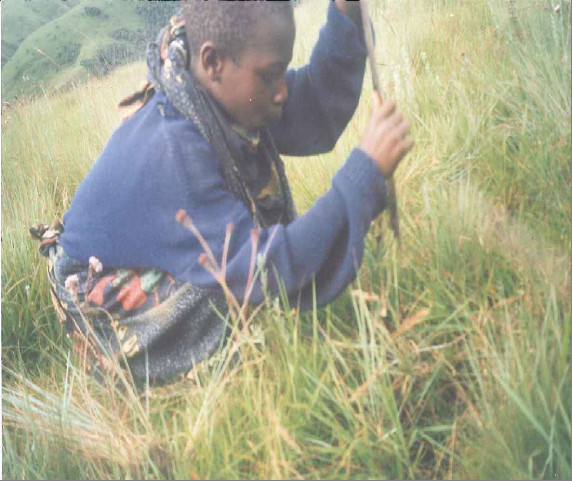
**AIDS orphan in the process of digging out an orchid tuber**.

Gathering households differ drastically with regard to their level of reliance on orchids (Table 2). Ninety-seven percent of HIV/AIDS affected households state that orchid gathering is their primary economic activity compared to non-HIV/AIDS affected households at 9.7 percent. Wild edible orchids are gathered by both HIV/AIDS and non-HIV/AIDS affected gatherers. The vast majority of adult gatherers, both among affected and non-affected households, are women (Figure 6). Among children, boys slightly outnumber girls among orphan gatherers and the opposite is true among non-affected children. When the sample based on HIV/AIDS is examined, the majority of gatherers are HIV/AIDS affected orphans (Figure 7 and Figure 8). Broken down by sex, the majority of gatherers are female (Table 3).

**Table 2:** Major economic activities for HIV/AIDS and non-HIV/AIDS households

Variable	Percentage of household HIV/AIDS affected (N = 100)	Percentage of household Non-HIV/AIDS affected (N = 52)
**Major economic activities**		
Agriculture production first, gathering activities a minor activity	3.0	90.3
Gathering orchids first, agriculture production comes second, third hired labour	29.0	9.7
Gathering orchids first and hired labour second	68.0	0.0
Total	100	100

**Table 3:** Description of wild orchid gatherer sample population

Categories of wild orchid gatherers	Number of respondents	Male (n = 70)	Female (n = 154)
Orphans HIV/AIDS affected	80	41	39
HIV/AIDS affected adults	31	1	30
Non-HIV/AIDS affected children	56	21	35
Non-HIV/AIDS affected adults	57	7	50

### Number of wild orchids known and number of gatherers in households

While the number of wild orchids mentioned in free listing from individual gathers ranged from one to seven species, the results from the Student's t-test indicate that there is no significant difference in the number of wild orchids known between HIV/AIDS and non-HIV/AIDS affected gatherers. Nor is there any significant difference in the number of gatherers in the two kinds of households. We do find, however, a difference in the frequency of gathering per week between HIV/AIDS affected gatherers and non-affected gatherers (Table 4).

**Table 4:** HIV/AIDS affected and non-affected comparison in gathering frequency, orchids known and number of household gatherers

	HIV/AIDS affected households (gatherers) (n = 111)	Non-HIV/AIDS affected households (gatherers) (n = 113)
**Variable**		
Gathering frequency	3.20 (1.9) *	2.25 (1.6) *
No. of wild orchid known	2.6 (0.9) ns	2.4 (0.9) ns
Number of gatherer in a household	2.9 (1.5) ns	3.3 (1.8) ns

* Significant at P = 0.05; number in parenthesis are standard deviations' means the mean difference is not significant; p > 0.05;number in parenthesis are standard deviations

### Frequency of gathering per week

Table 4 shows a significant difference in the frequency of gathering wild orchids per week between HIV/AIDS and non-HIV/AIDS affected gatherers, *t *(221) = 4.13, p = 0.00. That is, the mean number of days per week a HIV/AIDS affected individual gathers (M = 3.2, SD = 1.9) is significantly more than a non-HIV/AIDS affected gatherer (M = 2.25, SD = 1.6).

Further analysis (Table 5) using the ANOVA for multiple comparisons reveals that, within the HIV/AIDS affected category, there is no significant difference in gathering frequency between AIDS orphans and HIV/AIDS affected adults. However, there is a significant difference in gathering frequency between HIV/AIDS orphaned children, non-HIV/AIDS affected children and non-HIV/AIDS affected adults, with AIDS orphans gathering significantly more days per week. Among the non-HIV/AIDS gatherers, adults gathered significantly more days per week than children.

**Table 5:** Mean, standard deviation, and multiple comparison of gathering frequency among different categories of gatherers

Variables^a^	Mean and standard deviation^b^	Multiple comparison *p *value^c^
*Multiple comparison of gathering frequency between gatherers groups*		
		
***Orphans HIV/AIDS affected children***	3.61 (1.818)	
Comparison with non-affected children		0.000*
Comparison with HIV/AIDS affected adults		0.130 ns
Comparison with non-affected adults		0.002*
***HIV/AIDS affected adults***	2.84 (1.828)	
Comparison with non-affected adults		0.880 ns
Comparison with non-affected children		0.018*
		
***Non-HIV/AIDS affected children***	1.73 (1.070)	
Comparison with non-affected adults		0.044*
		
		
***Non-HIV/AIDS affected adults***	2.56 (1.852)	
Comparison with orphans HIV/AIDS affected children		0.002*
Comparison with HIV/AIDS affected adults		0.880 ns
Comparison with non-affected children		0.044*

^a^, variable subjected to multiple comparisons (orphan HIV/AIDS affected children; HIV/AIDS affected adults; non-HIV/AIDS affected and children and adults).

^b ^Mean and Standard deviation in gathering frequencies among gatherers categories

^c ^Multiple comparison between gatherers categories showing significance levels

### Gathering knowledge acquisition

The in-depth interviews reveal five self-reported sources of wild orchid gathering knowledge: from parents (mother), other child gatherers, middlemen/brokers, guardians, and fellow adult gatherers (Table 6). The majority (85%) of HIV/AIDS orphans acquired their gathering knowledge from their fellow child gatherers, while the lowest source was from middlemen/brokers (2.5%). Non-affected children, much like the orphans, more often acquired knowledge from their fellow child gatherers (62.5%), but unlike orphans, they also relied on obtaining knowledge from their mothers (37.5%). The majority of HIV/AIDS affected adults (54.8%) reported acquiring their gathering knowledge from their fellow adult gatherers followed by middlemen/brokers (45%). The non-affected adults primarily acquired their wild orchid gathering knowledge from their fellow adult gatherers (87.7%), and only 12.3% obtained their gathering knowledge from middlemen.

**Table 6:** Sources of orchid gathering knowledge acquisition

Gatherers categories	Mother	Other children gatherers	Middlemen/buyer/brokers	Guardians	Other adult gatherers
HIV/AIDS orphans (n = 80)	0	85	2.5	12.5	0
HIV/AIDS affected adults (n = 31)	0	0	45	0	54.8
Non-HIV/AIDS affected children (n = 56)	37.5	62.5	0	0	0
Non-HIV/AIDS affected adults (n = 57)	0	0	12.3	0	87.7

Further analysis reveals that HIV/AIDS affected gatherers differ significantly from non-HIV/AIDS affected gatherers in terms of gathering knowledge acquisition. A *chi square *test reveals that the source of gathering knowledge was significantly different for the two major categories of gatherers, HIV/AIDS and non-HIV/AIDS affected (*X*^2 ^= 61.846, P < 0.000, df = 4).

### Cognitive salience index (CSI) and Perception of edible wild orchid abundance

The cognitive salience index and knower's perception of wild orchid abundance is shown in Table 7. *Disa robusta *N.E. had the highest CSI (0.51), followed by *Satyrium atherstonei *Rchb.f (0.37), while *Eulophia schweinfrthii *Kraenzl and *Roeperocharis wentzeliana *Kraenzl had the lowest CSI (0.0045). The majority of gatherers who knew a given species had the perception that the species abundance was decreasing except for *Satyrium buchananii *(Table 7*)*.

**Table 7:** Cognitive Salience Index and gatherer's abundance assessments

		Knower's	Decreasing	Same	Increasing
		
Specie name	CSI	N	Percent^a^	Percent^a^	Percent^a^
*Disa robusta *N.E. Br.	0.51	74	79.7	20.3	0
*Satyrium atherstonei *Rchb. f.	0.37	106	83.0	14.2	2.8
*Habenaria xanthochlora *Schltr.	0.28	89	78.7	20.2	1.1
*Satyrium buchananii *Schltr.	0.25	119	37.8	39.5	22.7
*Disa erubescens *Rendle	0.24	57	96.0	3.5	0
*Eulophia schweinfurthii *Kraenzl	0.0045	1	100	0	0
*Roeperocharis wentzeliana *Kraenzl	0.0045	1	100	0	0

^a^, percent of knowers assessing abundance status, decreasing, same or increasing

## Discussion and Conclusions

HIV/AIDS orphans gather more frequently than any other category. HIV/AIDS affected adults follow in gathering frequency and lastly non-affected adults and non-affected children. There was no statistically significant difference between the number of gatherers in HIV/AIDS households and non-HIV/AIDS households, but the number of gatherers in both types of households is high. Taking the frequency of gathering into consideration and that multiple household members are engaged in gathering, the collection of edible orchid tubers appears to be an important activity for AIDS orphans and HIV/AIDS affected adults. There is a greater relative reliance on orchids among those affected by HIV/AIDS.

The significant difference in gathering frequency observed between AIDS orphans and others illustrates the precarious position of AIDS orphans. These children probably have a deep need to obtain cash income and must make their own way in the world. Gathering orchid tubers requires only the input of their own labor for a relatively high return, given the market demand for the edible orchids. More than two million orchid tubers find their way illegally to the markets of Zambia annually. This dependence on wild gathered products was anticipated in high prevalence HIV/AIDS communities. Increased dependency on wild gathered products usually follows a reduction of agricultural production or shortage of labor to perform farm activities [5,7]. In such rural contexts, affected households tend to increasingly depend on natural resources. However, considering the high market demand for edible orchid tubers, cash income may also be an incentive to gather among non-HIV/AIDS affected gatherers.

Local users' perceptions are essential in ascertaining species numbers and health where quantitative data on previous management outcomes (indicators) are not available [21]. Perceptions of resource abundance provide a potentially useful element in the planning of conservation programs and their assessment. While the majority of gatherers perceived six of the seven species to be in declining abundance, not all knower's had this perception. One aspect that may have a bearing on perceptions of abundance is the frequency with which an individual interacts with the resource. It is also expected that ethnobotanical knowledge is closely related to activities, and that those who interact often with the environment and a species will be more knowledgeable [22,23]. In this study, HIV/AIDS orphans have been shown to gather more frequently than any other category of gatherer, and naturally, one would expect the orphans to be more knowledgeable given the above. However, in the case of orphans, who acquire their gathering knowledge from other children, the acquisition of reliable gathering knowledge is questionable (including identification of the exact edible species). While there are numerous channels for knowledge acquisition, including direct experience, peers and grandparents, this knowledge can have shortcomings when parents are missing [24,25], particularly the mother [20,24,25]. Ultimately, knowledge of the useful environment comes not only by doing but by being taught directly.

Both HIV/AIDS affected and non-HIV/AIDS affected gatherers have overwhelming numbers of women gatherers. Very few men participate in gathering wild orchid tubers in the communities studied as most men are interested in timber and hence leave gathering orchids to children and women. All women gatherers in the HIV/AIDS affected category are widows, while women from the non-HIV/AIDS affected category are from dual-headed households (have a living male spouse with whom they reside). Gender and age based division of labor in gathering is noted for many culture areas [24-28].

A pattern emerges in the overall cognitive salience index of the seven gathered orchids. *Disa robusta *Rendle had the highest CSI (0.51), followed by *Satyrium atherstonei *Rchb.f., *Habenaria xanthochlora *Schltr, *Satyrium buchananni *Schltr, *Disa erubescence *Rendle. The lowest CSI (0.0045) was observed in *Eulophia scweinfurthii *Kraenzl and *Roeperocharis wentzeliana *Kraenzl. *Disa robusta *Rendle and *Satyrium atherstonei *Rchb.f had not only the highest CSI, but the majority of gatherers also knew the species and perceived it to be decreasing. The two species with both the lowest CSI and percentage of gatherers who free listed the orchids where *Eulophia scweinfurthii *Kraenzl and *Roeperocharis wentzeliana *Kraenzl.

Irrespective of age, most gatherers in this study perceived that marketable wild orchid species are no longer found in close proximity to their communities, but are very far away from their villages, a typical indication of declining abundance. This finding corresponds with a study in Uganda, where the gathering of non-timber forest products was one of the factors for abundance decline of non-timber forest products [29]. In the current study, once highly marketable orchids such as *Disa erubescence *Rendle and *Disa robusta *Rendle can no longer be easily found. As a result, orchid tubers considered to have inferior consumption quality (such as *Satyrium buchananii *Kranzl) are currently gathered. This finding agrees with what has been previously reported for Uganda [29], and the perception of a general decline in wild edible orchid abundance is in accordance with the quantitative botanical study conducted in this region [12]. This is not only a function of the high market demand from the Zambian market, but a function of the need to generate a living. HIV/AIDS increases reliance on the natural environment. This appears even more so for the most vulnerable, the AIDS orphaned children followed by HIV/AIDS widows.

It is our hope that in uncovering the differences in patterns between HIV/AIDS affected gatherers and non-affected gatherers we can ultimately make a contribution to understanding where future interventions might support people living with AIDS and the orchids.

## Competing interests

The authors declare that they have no competing interests.

## Authors' contributions

JFX-C collected the data in the three villages in the study area and analyzed the data under the supervision of LLP. JFX-C and LLP interpreted the data and drafted the framework for the paper and the discussion and conclusions. The writing of the article was a joint enterprise. All authors read and approved the final manuscript.
